# SARS-CoV-2 infection aggravates cigarette smoke-exposed cell damage in primary human airway epithelia

**DOI:** 10.1186/s12985-023-02008-z

**Published:** 2023-04-11

**Authors:** Rui Chen, Kenrie Pui-Yan Hui, Yingmin Liang, Ka-Chun Ng, John Malcolm Nicholls, Mary Sau-Man Ip, Malik Peiris, Michael Chi-Wai Chan, Judith Choi-Wo Mak

**Affiliations:** 1 Department of Medicine, School of Clinical Medicine, Li Ka Shing Faculty of Medicine, The University of Hong Kong, Hong Kong SAR, China; 2 Centre for Immunology and Infection, Hong Kong Science Park, Hong Kong SAR, China; 3 School of Public Health, Li Ka Shing Faculty of Medicine, The University of Hong Kong, Hong Kong SAR, China; 4 Department of Pathology, School of Clinical Medicine, Li Ka Shing Faculty of Medicine, The University of Hong Kong, Hong Kong SAR, China; 5 Department of Pharmacology & Pharmacy, Li Ka Shing Faculty of Medicine, The University of Hong Kong, Hong Kong SAR, China

**Keywords:** Cigarette smoking, COVID-19, SARS-CoV-2, Airway epithelial cells

## Abstract

**Background:**

The coronavirus disease 2019 (COVID-19), which is caused by severe acute respiratory syndrome coronavirus 2 (SARS-CoV-2), has become a worldwide pandemic with over 627 million cases and over 6.5 million deaths. It was reported that smoking-related chronic obstructive pulmonary disease (COPD) might be a crucial risk for COVID-19 patients to develop severe condition. As cigarette smoke (CS) is the major risk factor for COPD, we hypothesize that barrier dysfunction and an altered cytokine response in CS-exposed airway epithelial cells may contribute to increased SARS-CoV-2-induced immune response that may result in increased susceptibility to severe disease. The aim of this study was to evaluate the role of CS on SARS-CoV-2-induced immune and inflammatory responses, and epithelial barrier integrity leading to airway epithelial damage.

**Methods:**

Primary human airway epithelial cells were differentiated under air-liquid interface culture. Cells were then exposed to cigarette smoke medium (CSM) before infection with SARS-CoV-2 isolated from a local patient. The infection susceptibility, morphology, and the expression of genes related to host immune response, airway inflammation and damages were evaluated.

**Results:**

Cells pre-treated with CSM significantly caused higher replication of SARS-CoV-2 and more severe SARS-CoV-2-induced cellular morphological alteration. CSM exposure caused significant upregulation of long form angiotensin converting enzyme (ACE)2, a functional receptor for SARS-CoV-2 viral entry, transmembrane serine protease (TMPRSS)2 and TMPRSS4, which cleave the spike protein of SARS-CoV-2 to allow viral entry, leading to an aggravated immune response via inhibition of type I interferon pathway. In addition, CSM worsened SARS-CoV-2-induced airway epithelial cell damage, resulting in severe motile ciliary disorder, junctional disruption and mucus hypersecretion.

**Conclusion:**

Smoking led to dysregulation of host immune response and cell damage as seen in SARS-CoV-2-infected primary human airway epithelia. These findings may contribute to increased disease susceptibility with severe condition and provide a better understanding of the pathogenesis of SARS-CoV-2 infection in smokers.

**Supplementary Information:**

The online version contains supplementary material available at 10.1186/s12985-023-02008-z.

## Background

The ongoing worldwide pandemic of coronavirus disease 2019 (COVID-19), caused by severe acute respiratory syndrome coronavirus 2 (SARS-CoV-2), has had a devastating impact with over 627 million cases and over 6.5 million deaths [1]. The clinical feature of COVID-19 ranges from asymptomatic upper respiratory infection to severe pneumonia associated with acute respiratory distress syndrome [2]. However, the understanding of COVID-19 pathogenesis is still limited, it is an urgent need to identify the risk factors for worse outcomes.

Elderly COVID-19 patients and patients with comorbidities have higher risk suffering from poor prognosis and mortality [3]. Chronic obstructive pulmonary disease (COPD), as one of the leading causes of death globally, is a chronic inflammatory disease with slow progression and therefore the majority of patients are elderly [4]. Viral infection, as a comorbidity of COPD, increased the risk of severe exacerbations [5]. It was reported that COPD might be a crucial risk factor for COVID-19 patients, which intended to develop severe clinical outcome and increased in-hospital death [6, 7]. Cigarette smoke (CS), as the major risk factor for COPD, is a potential risk factor for COVID-19, given the high prevalence of use globally and its adverse effects on immune response resulting in higher susceptibility to most respiratory viruses [8]. CS was reported to have association with the negative progression and adverse effect of COVID-19, with an increasing risk of severe COVID-19 [9–11]. Patients with smoking history showed more severe symptoms under COVID-19 in comparison to the non-smokers [9].

Based on the knowledge that cigarette smoking predisposes to several respiratory infections and is associated with relatively poor outcomes in those suffering from these infection [12], the World Health Organization (WHO) released statements warning that smoking may increase the risk and severity of COVID-19 at the early stage of the pandemic [13]. However, many published studies have found low prevalence of active smokers among hospitalized patients with COVID-19 in different countries like China, France and USA [14–16]. Despite underrepresentation of smokers among patients with COVID-19, the association of smoking with clinical outcomes remains unclear. Usman and colleagues suggested that currently reported data were questionable due to heterogeneity of the studies and low liability of the data, and a protective effect of smoking should not be inferred [17].

It has been well-recognized that SARS-CoV-2 enters the cells via binding to the host receptor angiotensin-converting enzyme 2 (ACE2) [18], which is highly expressed on the apical surface of the airway epithelia of lung tissue [19]. Therefore, it is theoretically that increased ACE2 expression is likely correlated with increment of SARS-CoV-2 susceptibility. Currently, most studies showed upregulation of ACE2 expression after CS exposure and in smokers, while others showed no significant difference [20–24]. It was apparent that nicotine could reduce the ACE2 axis, which might be a potential pharmacological therapy for COVID-19 [25, 26]. Therefore, the consequence of CS on SARS-CoV-2-associated severe disease is still controversial.

As airway epithelium forms the first barrier against environmental attack, such as CS and viral infection, the epithelial barrier dysfunction has been suggested to be associated with the pathogenesis of COPD [27]. However, the role of airway epithelium in the pathogenesis of SARS-CoV-2 infection under smoking status remains unclear. We hypothesize that barrier dysfunction and an altered cytokine response in CS-exposed airway epithelial cells may contribute to increased SARS-CoV-2-induced immune response that may result in increased susceptibility to severe disease. In this study, we evaluated the effect of CS on the susceptibility of SARS-CoV-2 infection, SARS-CoV-2-induced cellular morphologic changes, host immune response, junctional disruption, mucus hypersecretion and ciliary disorder in well-differentiated primary normal human bronchial epithelial cells (HBECs) under air-liquid interface (ALI) culture.

## Methods

### Cigarette smoke medium (CSM) preparation

CS generated from two mouthpiece filter-removed cigarettes (Camel) was bubbled into 20 ml phosphate buffered saline and regarded as 100% CSM as previously described [28]. The CSM was filtered and standardized by measuring absorbance at a wavelength of 320 nm using a spectrophotometer (CLARIOstar®, BMG Labtech), as OD = 1.1.

### SARS-CoV-2 isolation

Vero E6 cells were used for virus isolation and propagation of SARS-CoV-2 (hCoV-19/Hong Kong/WHV-HK61-P3/2020, GenBank accession ID: OM403304). The original clinical specimens were collected from the nasopharyngeal and throat swab of a patient (young adult male) with confirmed COVID-19 in Hong Kong in January 2020 and isolated as previously described [29, 30]. Informed consent was obtained from the patient and approval was granted by the Institutional Review Board (IRB) of The University of Hong Kong and the Hospital Authority (Hong Kong West) (IRB approval no: UW 20–862).

### Cell culture

Normal primary HBECs (n = 5; Lonza) were seeded onto collagen-coated 12 mm transwell with 0.4 μm pore size and differentiated under ALI culture for 28 days in medium with 1:1 mixture of BEBM (Lonza) and DMEM (Gibco) supplemented with 52 µg/ml bovine pituitary extract, 5 µg/ml insulin, 10 µg/ml transferrin, 0.5 µg/ml hydrocortisone, 0.5 ng/ml human epidermal growth factor, 0.5 µg/ml epinephrine, 1.5 µg/ml bovine serum albumin and 15 ng/ml retinoic acid. Cells were maintained at 37 °C in a humidified 95% air/ 5% CO_2_ incubator. Passages 3 were used for experiments.

### CSM exposure and SARS-CoV-2 infection

After 18 hours’ starvation, the well-differentiated HBECs were treated with 2% CSM or medium only as control apically for 24 h. After CSM exposure, HBECs were infected with SARS-CoV-2 or mock infection (medium only as control) after thorough washing either at a multiplicity of infection (MOI) of 0.1 for viral replication kinetics or at a MOI of 2 for analysis of gene expression. Cell lysates were collected at 24- and 48-hours post-infection (hpi) for analysis of gene expression. Cells were fixed at 72 hpi in 2.5% glutaraldehyde or 10% formalin and processed for transmission electron microscopy (TEM) and immunohistochemistry (IHC) staining respectively (Additional File 1: Fig. S1).

### TEM imaging

Randomly selected ALI cultures of well-differentiated HBECs were fixed in 2.5% glutaraldehyde (Electron Microscopy Sciences) and sent to Electron Microscope Unit of The University of Hong Kong for further sample processing. The cells were observed under a Philips CM 100 transmission electron microscope.

### Semi-thin IHC staining

Resin-embedded samples were etched with 50% sodium ethoxide and graded ethanol. After blocking with 3% H_2_O_2_, the slides were blocked with 2% normal horse serum and incubated with primary antibody against SARS-CoV-2 Nucleoprotein (#40,143-T62, Sino Biological, 1:100 dilution). Followed by Impress HRP Horse anti-Rabbit IgG (Vector Labs), the sections were developed with ImmPACT NovaRED Substrate Kit (Vector Labs) and counterstained with toluidine blue.

### RNA extraction and RT-PCR

Well-differentiated HBECs were harvested for RNA extraction using MiniBEST Universal RNA extraction kit (Takara) and reverse transcription of RNA was conducted with EvoScript Universal cDNA Master kit (Roche). Quantitative RT-PCR assay was performed using SYBR Green Real-Time Master Mix (Applied Biosystems). All procedures were conducted following the manuals provided by the manufacturer. Relative quantity of mRNA was obtained by using the comparative *Ct* method and normalized by housekeeping genes such as human ribosomal protein S13 as previously described [31]. The primers used are listed in Additional File 1: Table S1.

### Cytokine assay

Cell culture supernatants from the apical surface of the cells were collected at 24 and 48 hpi to detect the protein concentration of interleukin (IL)-6 using Cytometric Bead Array (BD Bioscience) according to the manufacturer’s instruction. The level of IL-6 was analyzed using FCAP Array Analysis Software (BD Bioscience).

### Statistical analysis

Data were presented as mean ± standard error of mean (SEM). One-way ANOVA test with post hoc analysis (Tukey) was applied to compare multiple groups. Student’s *t*-test was also implemented for variables measured at a single time point where appropriate. All the statistical analyses were performed using GraphPad Prism 7 (GraphPad Software). Significance was achieved if *p* value was less than 0.05.

## Results

### Effect of CS on SARS-CoV-2 infection morphology and susceptibility

**Fig. 1:**
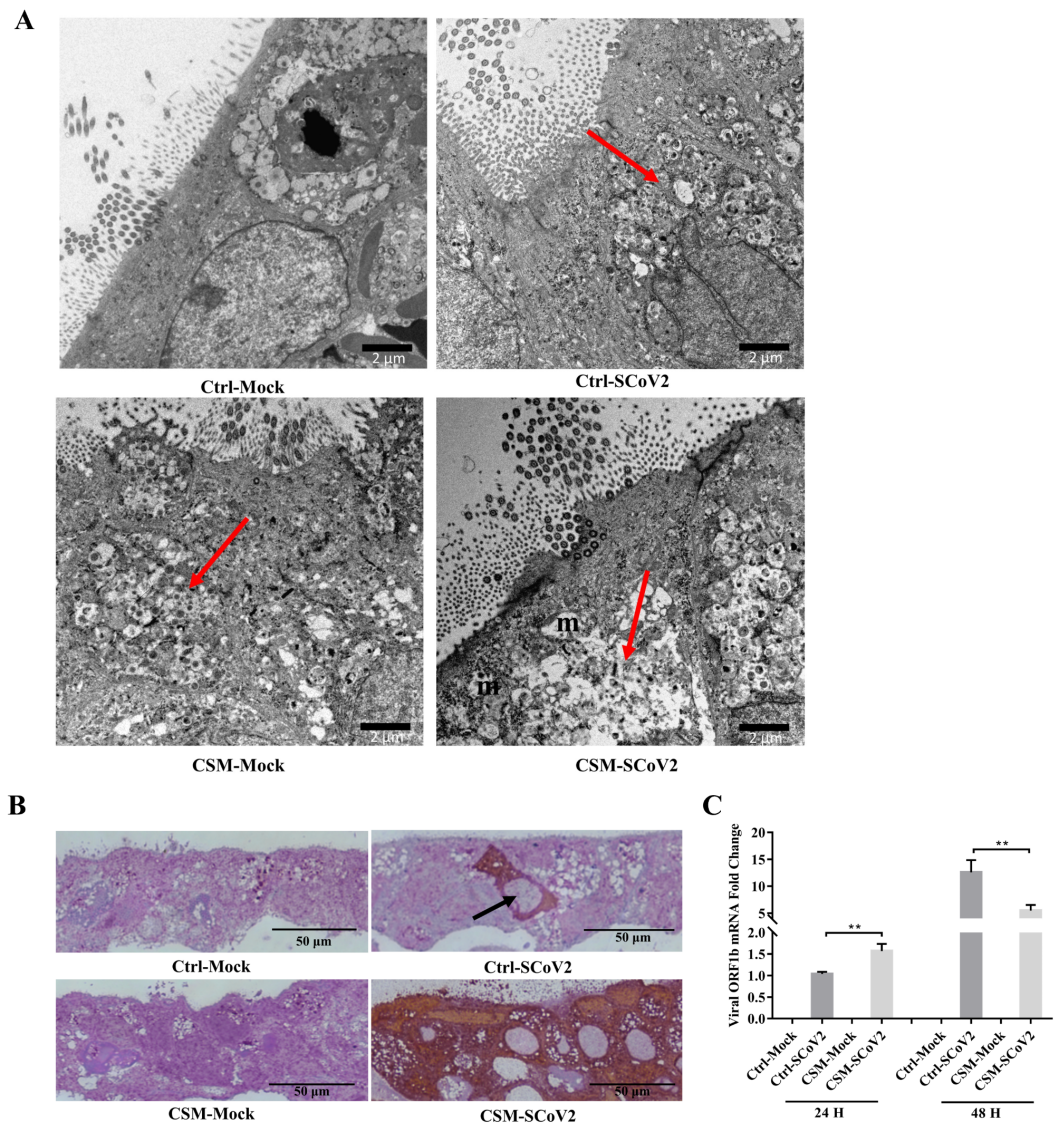
Effect of cigarette smoke on SARS-CoV-2-induced morphological change and susceptibility to SARS-CoV-2 infection in airway epithelial cells. **A** Representative transmission electron micrographs of well-differentiated normal human bronchial epithelial cells (MOI = 0.1). m: mitochondria; vesicles are red arrows pointed. Scale bar, 2 μm. **B** Representative images of immunohistochemical staining with a polyclonal antibody against the SARS-CoV-2 nucleoprotein protein (MOI = 0.1). Positive cells are red-brown (black arrows). Scale bar, 50 μm. **C** mRNA expression of viral gene ORF1b (MOI = 2). Values are expressed as mean ± SEM (n = 5). ^**^p < 0.01 for unpaired Student’s *t*-test. Ctrl, control; CSM, cigarette smoke medium; SCoV2, SARS-CoV-2; H, hours

More severe cell damages induced by SARS-CoV-2 infection were observed after CSM exposure (Fig. 1A) examined by TEM, in which swollen vesicles were observed. SARS-CoV-2 infection caused an increase in the number of enlarged vesicles (Fig. 1A). Degeneration of mitochondria induced by SARS-CoV-2 was also found after CSM exposure (Fig. 1A). Despite no significant difference on replication kinetics measured by 50% tissue culture infectious dose (TCID_50_) titrations from the apical washing of the well-differentiated epithelial cells (Additional File 1: Fig. S2), the IHC staining for SARS-CoV-2 nucleoprotein showed higher number of cells with positive staining for viral antigen (red-brown) in the well-differentiated epithelial cells after CSM exposure at MOI 0.1, suggesting an increment in viral susceptibility (Fig. 1B). We further measured the viral gene ORF-1b expression at 24, 48 hpi at MOI 2. The expression of ORF-1b was significantly higher after CSM exposure at 24 hpi. On the contrary, the expression of ORF-1b on CSM-exposed cells was significantly lower than infection alone at 48 hpi, suggesting a rapid effect of CS on SARS-CoV-2 infection which might be due to the toxicity of infection on the host cells (Fig. 1C).

### Effect of CS on ACE2 and serine protease

**Fig. 2:**
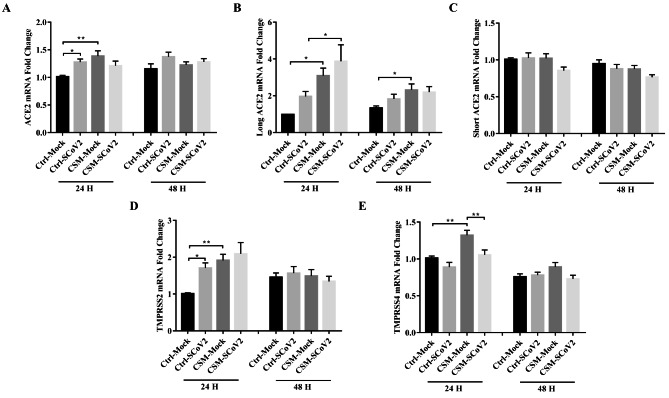
Effect of cigarette smoke on SARS-CoV-2-induced ACE2 and serine protease expression. **A** mRNA expression of total ACE2. **B** mRNA expression of long form ACE2. **C** mRNA expression of short form ACE2. **D** mRNA expression of TMPRSS2. **E** mRNA expression of TMPRSS4. Ctrl, control; CSM, cigarette smoke medium; SCoV2, SARS-CoV-2; H, hours. MOI of 2 was used for analysis of gene expression. Values are expressed as mean ± SEM (n = 5). ^*^p < 0.05, ^**^p < 0.01 for One-way ANOVA test with post hoc analysis and Tukey correction

ACE2, as previously described, is the binding receptor for SARS-CoV-2. We showed that the total ACE2 mRNA expression was upregulated after CSM exposure at 24 hpi but not at 48 hpi with no significant effects upon SARS-CoV-2 infection (Fig. 2A). ACE2 has recently been sub-categorized into short and long forms in the airway epithelium, in which the short form ACE2 was not able to bind the virus due to the lack of SARS-CoV-2 spike high-affinity binding sites [32]. Our results showed that, the expression of long form ACE2 mRNA was significantly higher after CSM exposure without affecting infection at both 24 and 48 hpi, and was further upregulated after SARS-CoV-2 infection at 24 hpi (Fig. 2B). On the contrary, no significant difference on short form ACE2 mRNA expression was observed after CSM exposure at both time points (Fig. 2C). Transmembrane serine protease (TMPRSS) 2, which cleaves the spike protein of SARS-CoV-2, promotes the entry of virus into the cells [18]. CSM exposure or SARS-CoV-2 infection itself upregulated the expression of TMPRSS2 mRNA at 24 hpi. A trend of further induction was observed after viral infection in CSM-exposed cells. However, there were no significant differences among different groups at 48 hpi (Fig. 2D). The significance of TMPRSS4 with similar function as TMPRSS2 in viral infection, has been suggested in smokers and its relation to COVID-19 [20]. The expression of TMPRSS4 mRNA was elevated after CSM exposure which was suppressed by SAR-CoV-2 infection at 24 hpi. No significant differences among different groups were observed at 48 hpi (Fig. 2E).

### Effect of CS on SARS-CoV-2-induced immune response

**Fig. 3:**
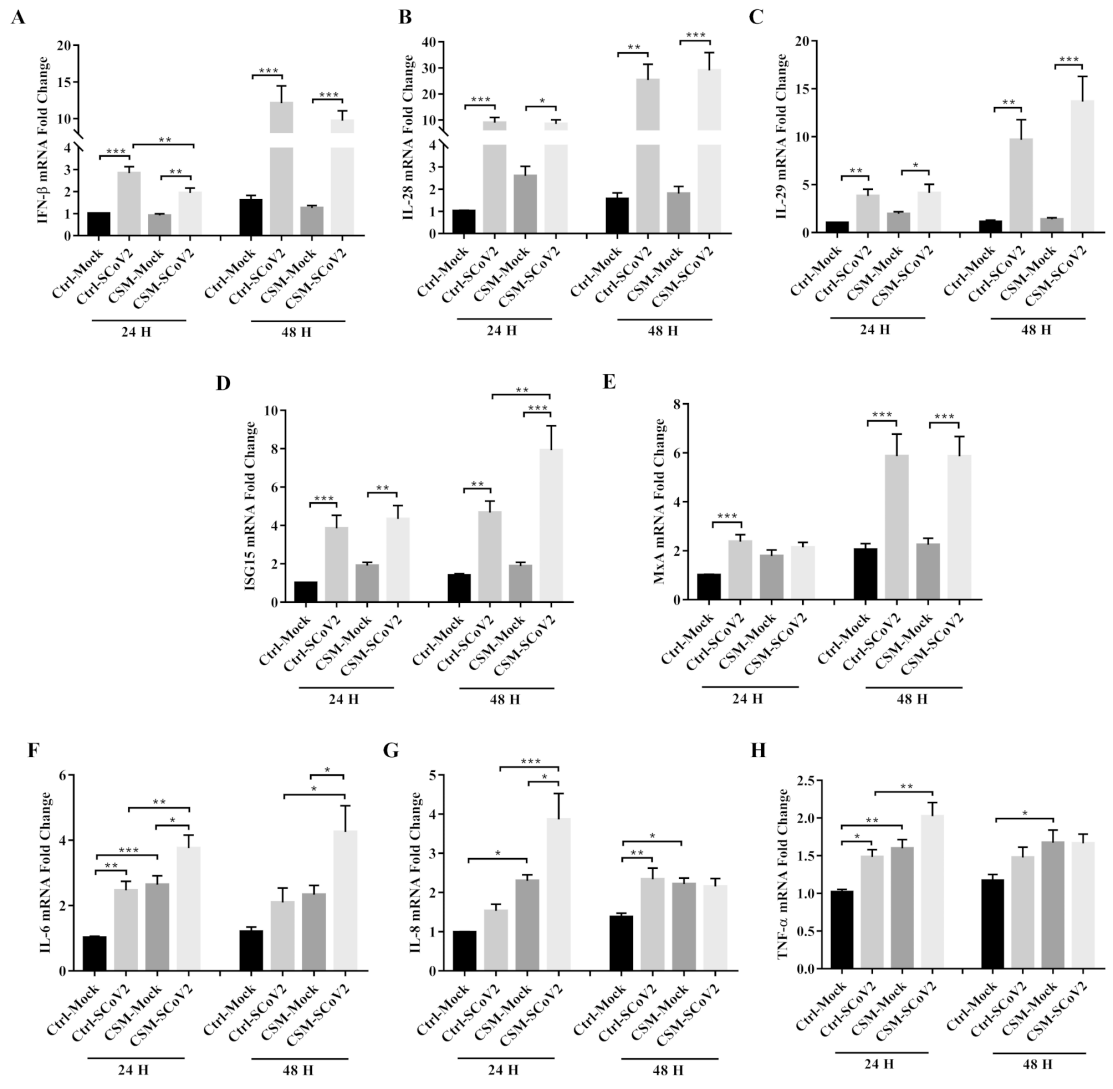
Effect of cigarette smoke on SARS-CoV-2-induced immune response. **A** mRNA expression of IFN-β. **B** mRNA expression of IL-28. **C** mRNA expression of IL-29. **D** mRNA expression of ISG15. **E** mRNA expression of MxA. **F** mRNA expression of IL-6. **G** mRNA expression of IL-8. **H** mRNA expression of TNF-α. Ctrl, control; CSM, cigarette smoke medium; SCoV2, SARS-CoV-2; H, hours. MOI of 2 was used for analysis of gene expression. Values are expressed as mean ± SEM (n = 5). ^*^p < 0.05, ^**^p < 0.01, ^***^p < 0.001 for One-way ANOVA test with post hoc analysis and Tukey correction

Type I interferon (IFN), IFN-β mRNA expression was significantly upregulated after SARS-CoV-2 infection at both 24 and 48 hpi, while CSM-treated cells inhibited SARS-CoV-2-induced IFN-β mRNA expression at 24 hpi (Fig. 3A). Type III IFNs, IL-28 and IL-29 mRNA expressions were also significantly increased by SARS-CoV-2 infection at both 24 and 48 hpi, while no significant effect of CSM on SARS-CoV-2-induced IL-28 and IL-29 mRNA expressions were observed (Fig. 3B and C). The expressions of interferon-stimulated gene 15 (ISG15) and myxovirus resistance protein 1 (MxA) mRNA, which are essential in the host antiviral response, were upregulated significantly after SARS-CoV-2 infection at both 24 and 48 hpi, while CSM exposure further upregulated SARS-CoV-2-induced ISG15 mRNA expression at 48 hpi (Fig. 3D and E). Inflammatory cytokine genes, IL-6, IL-8 and tumor necrosis factor α (TNF-α) mRNA expressions were elevated after SARS-CoV-2 infection or CSM exposure. However, CSM-exposed cells further upregulated SARS-CoV-2-induced expressions of inflammatory cytokine genes at 24 hpi. The IL-6 mRNA expression was sustained after SARS-CoV-2 infection in CSM-exposed cells at 48 hpi (Fig. 3, F-H), in line with the release of IL-6 at the apical side of the supernatants collected from cell cultures (Additional File 3: Fig. S3).

### Effect of CS on SARS-CoV-2-induced tight junction disruption and mucus hypersecretion

**Fig. 4:**
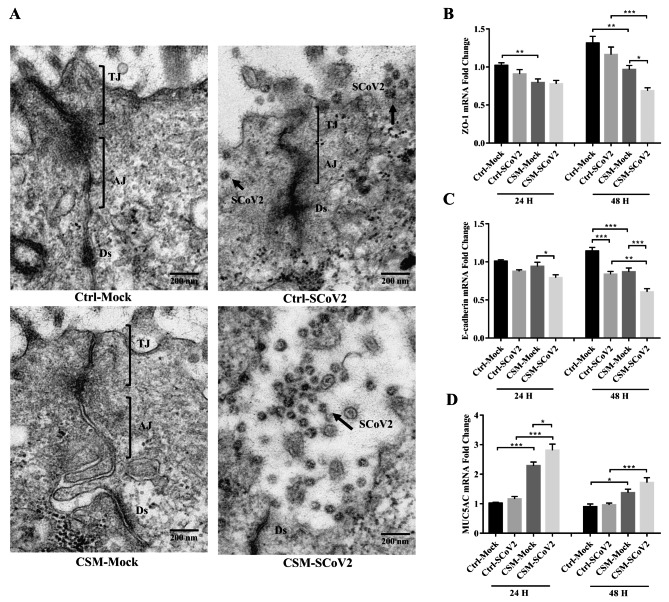
Effect of cigarette smoke on SARS-CoV-2-induced tight junction’s disruption and mucus hypersecretion. **A** Representative transmission electron micrographs of the tight junctions in well-differentiated airway epithelial cells (MOI = 0.1). Scale bar, 200 nm. **B** mRNA expression of ZO-1. **C** mRNA expression of E-cadherin. **D** mRNA expression of MUC5AC. TJ, tight junctions; AJ, adherens junctions; Ds, desmosomes; Ctrl, control; CSM, cigarette smoke medium; SCoV2, SARS-CoV-2; H, hours. MOI of 2 was used for analysis of gene expression. Values are expressed as mean ± SEM (n = 5). ^*^p < 0.05, ^**^p < 0.01, ^***^p < 0.001 for One-way ANOVA test with post hoc analysis and Tukey correction

Normally, the well-differentiated epithelial cells were connected by junctional complexes, including tight junctions, adherens junctions and desmosomes, forming a tightly packed epithelium without intercellular spaces [33]. Our TEM results showed that after CSM exposure, SARS-CoV-2 infection completely broke down the tight junctions and adherens junctions complex and entered the cells (Fig. 4A). This was further confirmed by detecting tight junction marker, zonula occluden-1 (ZO-1), and adherens junction marker, E-cadherin. In CSM-exposed cells, SARS-CoV-2 infection further downregulated the expressions of ZO-1 and E-cadherin mRNA at 48 hpi (Fig. 4B and C). Mucin gene marker, mucin 5AC (MUC5AC) mRNA expression was upregulated only after CSM exposure, which was further induced by SARS-CoV-2 infection at 24 hpi and 48 hpi to a smaller extent (Fig. 4D).

### Effect of CS on SARS-CoV-2-induced ciliary disorder

**Fig. 5:**
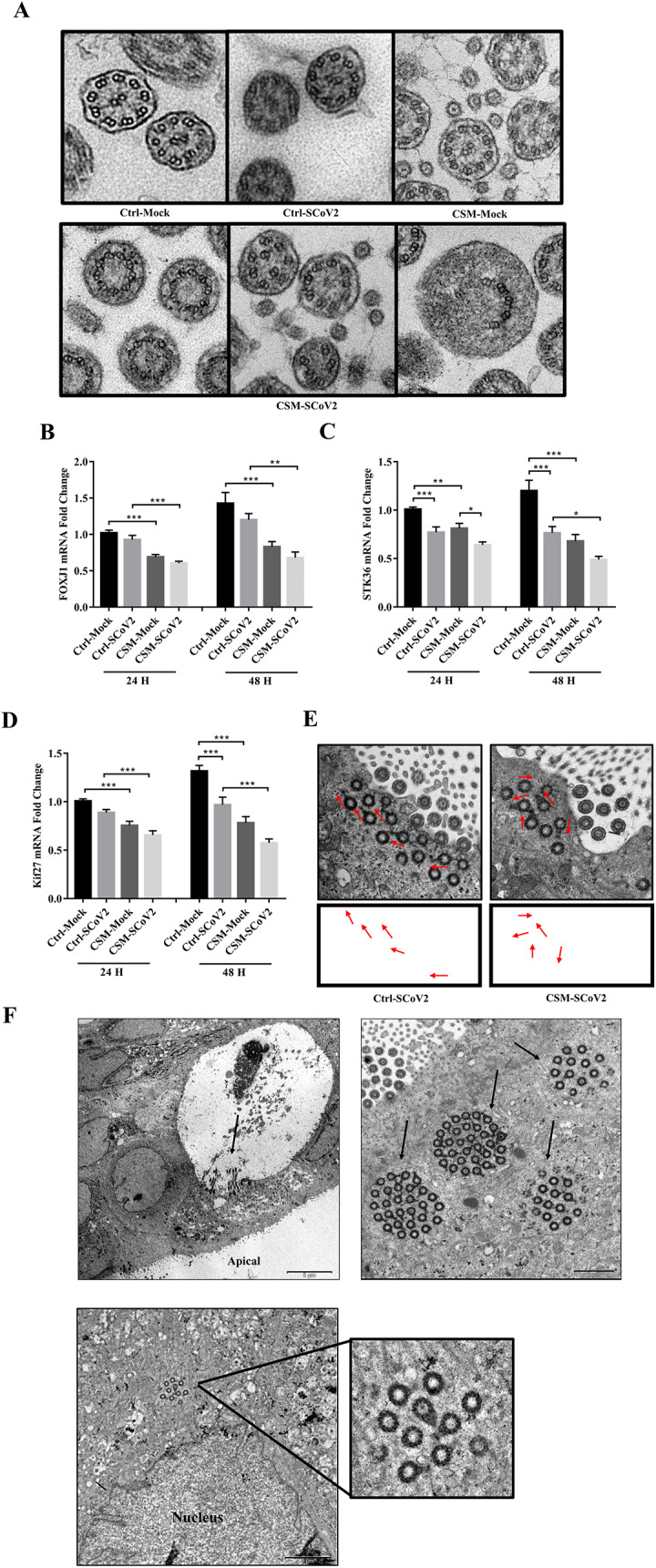
Effect of cigarette smoke on SARS-CoV-2-induced motile ciliary disorder. **A** Representative transmission electron micrographs of motile cilia axonemes. **B** mRNA expression of FOXJ1. **C** mRNA expression of STK36. **D** mRNA expression of Kif27. **E** Representative transmission electron micrographs of basal body polarity (red arrows). **F** Representative transmission electron micrographs of cilia regeneration were shown. Ctrl, control; CSM, cigarette smoke medium; SCoV2, SARS-CoV-2; H, hours. MOI of 0.1 and MOI of 2 were used for analysis of transmission electron micrographs and gene expression respectively. Values are expressed as mean ± SEM (n = 5). ^*^p < 0.05, ^**^p < 0.01, ^***^p < 0.001 for One-way ANOVA test with post hoc analysis and Tukey correction

Functional motile cilia were normally composed by nine peripheral microtubule doublets surround a central pair of singlet microtubules [i.e., a (9 + 2) axoneme, refer to Fig. 5A Ctrl-Mock group] [34]. After SARS-CoV-2 infection with CSM-exposed cells, abnormal ciliary ultrastructure was observed, with lack and misposition of central pair apparatus and swollen motile cilia (Fig. 5A CSM-SCoV2 group). To further confirm the results, we detected the motile ciliary marker (Forkhead box J1, FOXJ1), central pair apparatus marker (serine/threonine kinase 36, STK36) and basal body marker (kinesin-like protein 27, Kif27) for controlling the cilia orientation [34, 35]. All the ciliary markers were downregulated by CSM exposure at both 24 and 48 hpi. SARS-CoV-2 infection only caused reduction in STK36 mRNA expression at both 24 and 48 hpi, and Kif27 mRNA expression at 48 hpi but not FOXJ1 mRNA expression at both 24 and 48 hpi (Fig. 5B-D). However, prior CSM exposure further downregulated SARS-CoV-2-supressed expressions of FOXJ1 and Kif27 mRNA at both 24 and 48 hpi as well as STK36 mRNA expression only at 48 hpi (Fig. 5B-D). We also measured the cilia polarity by labelling the polar orientation of basal body under TEM. For the SARS-CoV-2 infection alone, the basal body polarity aligned with each other properly, but after CSM exposure and then viral infection, the basal body polarity was misaligned and was observed in antiparallel orientations (Fig. 5E). In addition, regeneration of new ciliated cells grown from a vacuolar structure and the basal body close to the nucleus in the existing cells of the motile cilia were novelly found in CSM-exposed cells after SARS-CoV-2 infection (Fig. 5F).

## Discussion

In this study, well-differentiated primary HBECs were treated with CSM before SARS-CoV-2 infection. The findings demonstrated an increase in viral replication rapidly after CSM exposure with an increase in severity of cell damage and number of infected cells. The rapid effect of CS was in line with our findings on viral binding receptors ACE2 and TMPRSS. The SARS-CoV-2 infection of airway epithelial cells involves the binding of SARS-CoV-2 spike protein into ACE2 receptors following a protease-mediated activation of membrane fusion via endocytic machinery to promote viral replication [36]. In a previous study, direct CS exposure on airway epithelium increased the number of SARS-CoV-2 infected cells, in line with the current finding that a rapid effect of CS on SARS-CoV-2 infection [21]. In support, a single-cell RNA sequencing study showed that bronchial epithelial cells from COPD patients with smoking as the most important risk factor are more susceptible to SARS-CoV-2 infection with increasing expression of coreceptor [37]. However, there was controversial findings on ACE2 expression and CS exposure [23, 38]. Our results showed an upregulation of total ACE2 expression at early time point after CSM exposure, however, no effect on SARS-CoV-2-induced total ACE2 expression was observed. Recently, ACE2 was subcategorized into short form and long form ACE2, in which short forms ACE2 were incapable of binding the SARS-CoV-2 spike protein due to the lack of high-affinity binding sites [32]. This study showed that CSM exposure elevated SARS-CoV-2-induced long form ACE2 expression as well as TMPRSS2 and TMPRSS4 expression at the early time point, indicating that CSM exposure might facilitate the virus entry with rapid onset.

The IFN response is the major first line of defense against viruses. Viral infections preferentially activate type I and type III IFN response [39]. Though type I and type III IFNs induce similar ISGs downstream, type I IFN signaling results in a more rapid induction and declines of ISG expressions [39, 40]. When there is a lack of IFN response to control the initial viral replication, the late onset IFN response may result in severe inflammation. Our data showed a delayed type I IFN response induced by SARS-CoV-2 after CSM exposure at early time point, suggesting that CSM might inhibit SARS-CoV-2-induced type I IFN response. CSM also increased the expression of SARS-CoV-2-induced inflammatory cytokines such as IL-6, which might further lead to worsening outcome. Therefore, CSM might lead to an aggravated inflammation via inhibition of type I IFN signaling pathway and type I IFNs might be a potential therapy for COVID-19 patients [41], especially in smokers.

The apical junctional complex of airway epithelial cells is essential and creates the barrier for viral invasion, which consist of tight and adherens junctions, and desmosomes [42]. One previous study suggested that SARS-CoV-2 infection led to disruption of tight junctions by downregulation of ZO-1[43], in line with our current findings. We found downregulation of both tight junction marker (ZO-1) and adherens junction marker (E-cadherin) after SARS-CoV-2 infection, and CSM further attenuated the expression of both markers. However, our TEM results show a complete disruption of the junctional complex only in the SARS-CoV-2-infected cells after prior CSM exposure suggesting the irreversible effect of CSM on SARS-CoV-2-induced junctional disruption. Mucus hypersecretion, a key pathophysiological characteristic of COPD, is a feature of chronic bronchitis, which causes chronic productive cough and expectoration [44]. It is well known that mucus hypersecretion increases the risk of severe COPD exacerbation due to bacterial or viral infection [45]. Mucus is mainly produced by goblet cells at airway epithelial surface, and MUC5AC, is the major gel-forming mucin, which is the predominant subtype found in COPD patients [46]. Elevation of MUC5AC level was associated with COPD initiation, progression, exacerbation risk and the overall pathogenesis, which suggested that MUC5AC might be a novel biomarker for COPD prognosis [47]. In agreement with the previous studies [48, 49], CSM exposure induced MUC5AC overexpression with further upregulation of MUC5AC expression after SARS-CoV-2 infection, suggesting that SARS-CoV-2 might aggravate mucus hypersecretion to cause further airway obstruction in smokers and COPD patients. Epithelial cilia are the first line of defense against virus invasion and mucociliary clearance, which play an important role for many airway diseases including COPD [50, 51]. The motile ciliary disorder was identified by abnormal ciliogenesis, ciliary ultrastructure and dysfunction of ciliary motility [52]. Our results demonstrated dysfunction of ciliary motility through significant downregulations of motile ciliary marker, central pair apparatus marker and basal body marker as well as the cilia orientation after CSM exposure and SARS-CoV-2 infection. TEM data showed the abnormality in ciliary ultrastructure, with missing and misposition of central pair apparatus and swollen cilia. In this study, novel findings on the regeneration of new ciliated cells grown from a vacuolar structure and the basal body close to the nucleus in the existing cells of motile cilia, suggesting the abnormality in ciliogenesis. Taken together, CSM aggravated SARS-CoV-2-induced severe motile ciliary disorders.

However, this study is not without limitations, exposure of CSM overnight is unlikely to be presentative of the effects that may be caused by the history of pack-year smoking in human. Due to technical difficulties, we treated the cells with CSM on the apical side of the well-differentiated HBECs instead of direct CS exposure. Besides, the composition of CSM in soluble fraction and the loss of volatile components are unknown which may affect the results. For example, nicotine, as one of the components in CS, may even be able to prevent and treat COVID-19 due to its anti-inflammatory effects [26]. Furthermore, our study focuses only on HBECs using the in vitro model. Airway epithelial cells may interact with infiltrating inflammatory cells such as neutrophils or macrophages, which will need further investigation to elucidate the mechanism on cell-cell interaction during SARS-CoV-2 infection using co-culture in vitro model. Finally, future studies with CS-exposed passive smoking rodent model should be performed to investigate the effects of SARS-CoV-2 infection on CS-induced airway cell damage in vivo.

## Conclusion

**Fig. 6:**
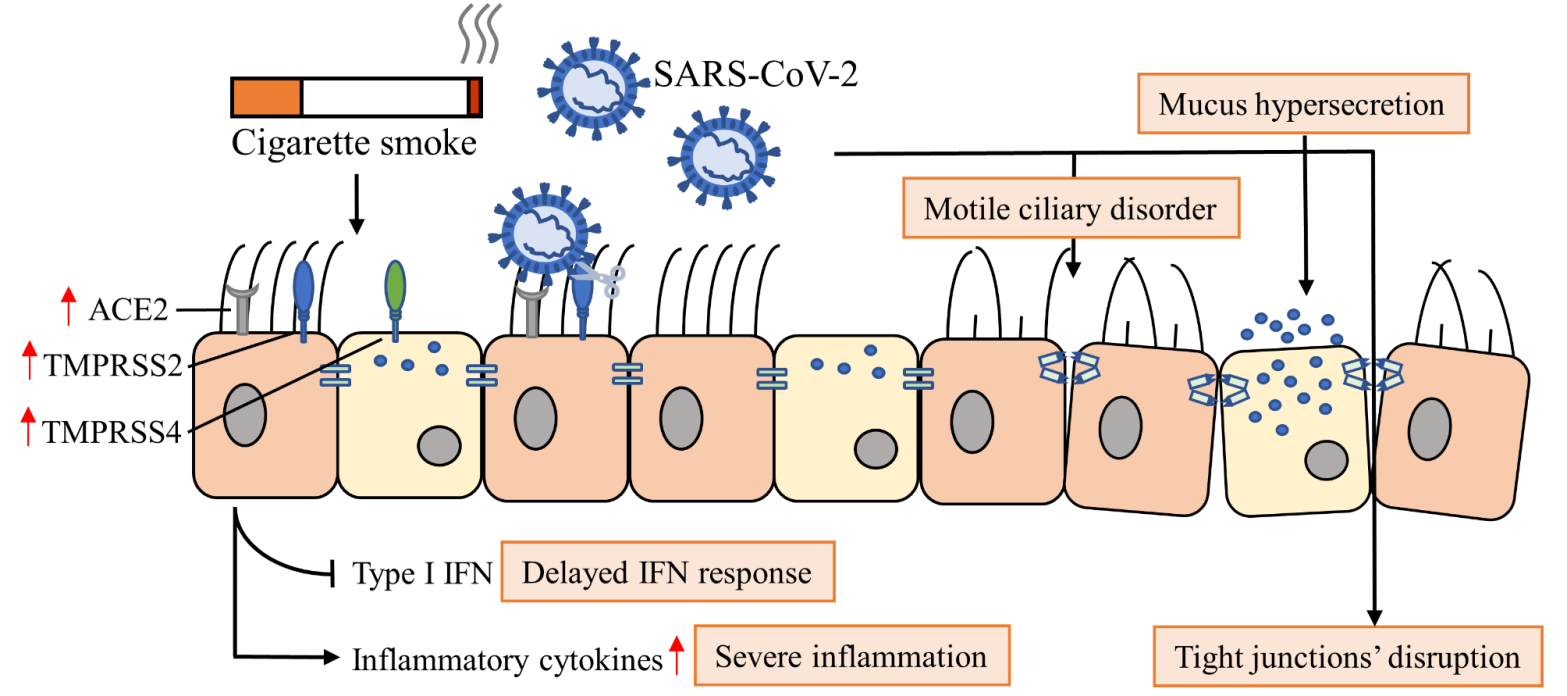
Proposed schematic diagram showing the effect of cigarette smoke on airway epithelium in the pathogenesis of SARS-CoV-2-infection.

Despite contradictory findings among reports about whether CS exposure increases SARS-CoV-2 susceptibility and results in worsening clinical outcomes, this study reported findings that in well-differentiated primary HBECs, treatment with CSM before SARS-CoV-2 infection caused an increase in viral replication with an increase in severity of cell damage and number of infected cells, upregulation of long form ACE2, TMPRSS2 and TMPRSS4 that might contribute to the viral entry. In addition, delayed type I IFN response was observed, resulting in insufficient IFNs to control the initial viral replication and led to more severe inflammation. CSM exposure worsened SARS-CoV-2-induced airway epithelial cell damage, leading to the disruption of epithelial tight/adherens junctions, mucus hypersecretion and severe motile ciliary disorder (Fig. 6).

Taken together, the current findings from multiparameter analysis of HBECs point to support that smoking upregulates the ACE2 as binding receptor for SARS-CoV-2 and TMPRSS2/4 as helping the viral entry, leading to an aggravated host immune response via inhibition of type I interferon pathway. Smoking also worsens SARS-CoV-2-infected airway epithelial cell damage through junctional disruption of the integrity of tight junction barrier, mucus hypersecretion and severe motile ciliary disorder. Therefore, the current findings may contribute to increased disease susceptibility with severe condition. This study has greatly increased our understanding of the pathogenesis of COVID-19-associated airway epithelial damage in smokers via multifactorial mechanisms.

## Electronic supplementary material

**Fig. S1** Experimental protocol for in vitro model. Schematic overview of air-liquid interface (ALI) culture of well-differentiated HBEC model with different treatments (n=5). **Fig. S2** Effect of cigarette smoke on viral replication kinetics in airway epithelial cells. Data were measured by TCID_50_ assay. Ctrl, control; CSM, cigarette smoke medium; SCoV2, SARS-CoV-2. Values are expressed as mean ± SEM (n=5). **Fig. S3** Effect of cigarette smoke on SARS-CoV-2-induced cytokine IL-6 release at the apical side of the supernatants collected from cell cultures. Ctrl, control; CSM, cigarette smoke medium; SCoV2, SARS-CoV-2; H, hours. Values are expressed as mean ± SEM (n=5). *p<0.05 for One-way ANOVA test with post hoc analysis and Tukey correction. **Table S1** Quantitative PCR primer sequences.

## Data Availability

The datasets used and/or analysed during the current study are available from the corresponding authors on reasonable request.

## List of abbreviations

ACE2: Angiotensin-converting enzyme 2
ALI: Air-liquid interface
COPD: Chronic obstructive pulmonary disease
COVID-19: Coronavirus disease 2019
CS: Cigarette smoke
CSM: Cigarette smoke medium
FOXJ1: Forkhead box J1
HBEC: Human bronchial epithelial cell
hpi: Hours post-infection
IFN: Interferon
IL: Interleukin
ISG15: Interferon-stimulated gene 15
Kif27: Kinesin-like protein 27
MOI: Multiplicity of infection
MUC5AC: Mucin 5AC
MxA: Myxovirus resistance protein 1
SARS-CoV-2: Severe acute respiratory syndrome coronavirus 2
SEM: Standard error of mean
STK36: Serine/threonine kinase 36
TCID_50_: 50% Tissue culture infectious dose
TEM: Transmission electron microscopy
TMPRSS: Transmembrane serine protease
TNF-α: Tumor necrosis factorα
ZO-1: Zonula occluden-1
