# Supplementary material for: SARS-CoV-2 infection aggravates cigarette smoke-exposed cell damage in primary human airway epithelia
Source: Virol J. 2023 Apr 11;20:65. doi: 10.1186/s12985-023-02008-z (PMC10089376; doi:10.1186/s12985-023-02008-z)
Supplement: Supplementary file 1 — Fig. S1 Experimental protocol for in vitro model. Schematic overview of air-liquid interface (ALI) culture of well-differentiated HBEC model with different treatments (n=5). Fig. S2 Effect of cigarette smoke on viral replication kinetics in airway epithelial cells. Data were measured by TCID50 assay. Ctrl, control; CSM, cigarette smoke medium; SCoV2, SARS-CoV-2. Values are expressed as mean ± SEM (n=5). Fig. S3 Effect of cigarette smoke on SARS-CoV-2-induced cytokine IL-6 release at the apical side of the supernatants collected from cell cultures. Ctrl, control; CSM, cigarette smoke medium; SCoV2, SARS-CoV-2; H, hours. Values are expressed as mean ± SEM (n=5). *p<0.05 for One-way ANOVA test with post hoc analysis and Tukey correction. Table S1 Quantitative PCR primer sequences. [file 12985_2023_2008_MOESM1_ESM.docx]

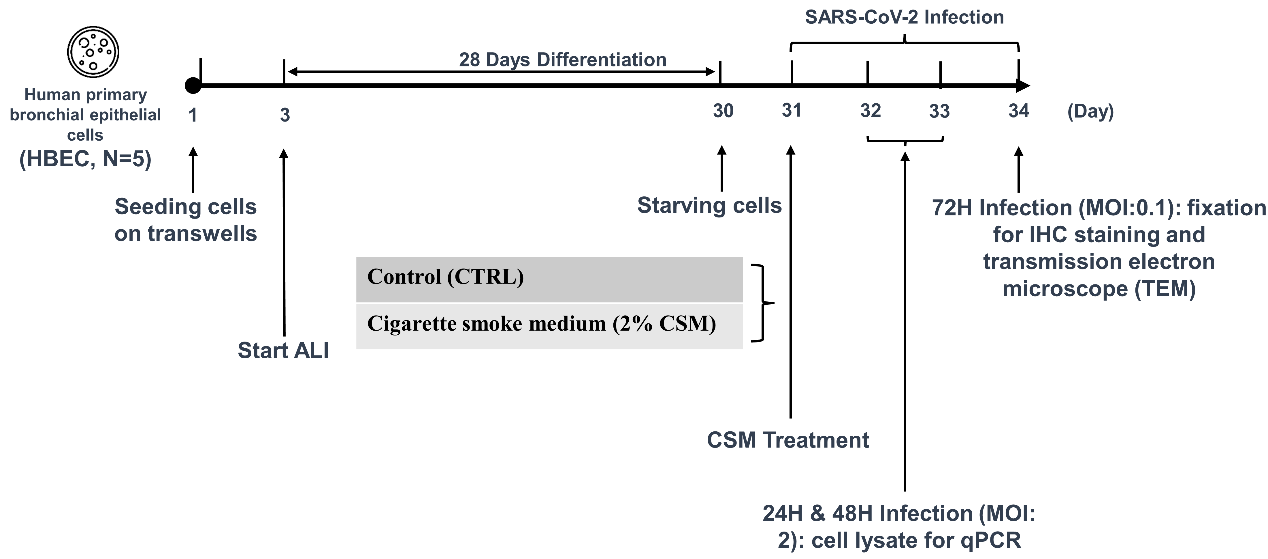


**Fig. S1** Experimental protocol for *in vitro* model. Schematic overview of air-liquid interface (ALI) culture of well-differentiated HBEC model with different treatments (n=5).


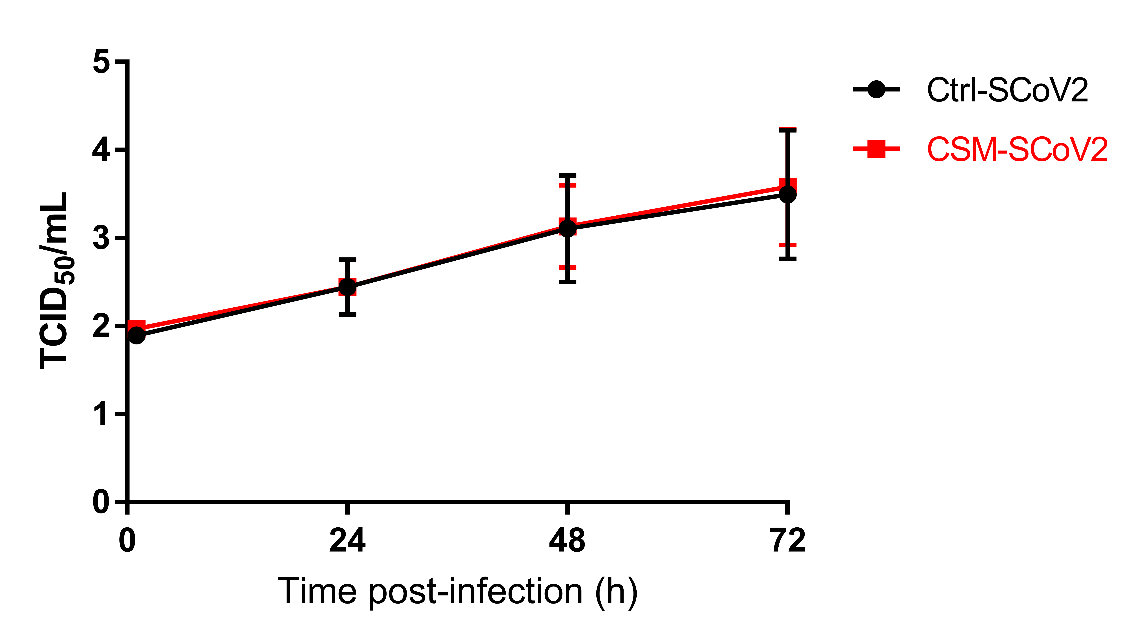


**Fig. S2** Effect of cigarette smoke on viral replication kinetics in airway epithelial cells. Data were measured by TCID_50_ assay. Ctrl, control; CSM, cigarette smoke medium; SCoV2, SARS-CoV-2. Values are expressed as mean ± SEM (n=5).


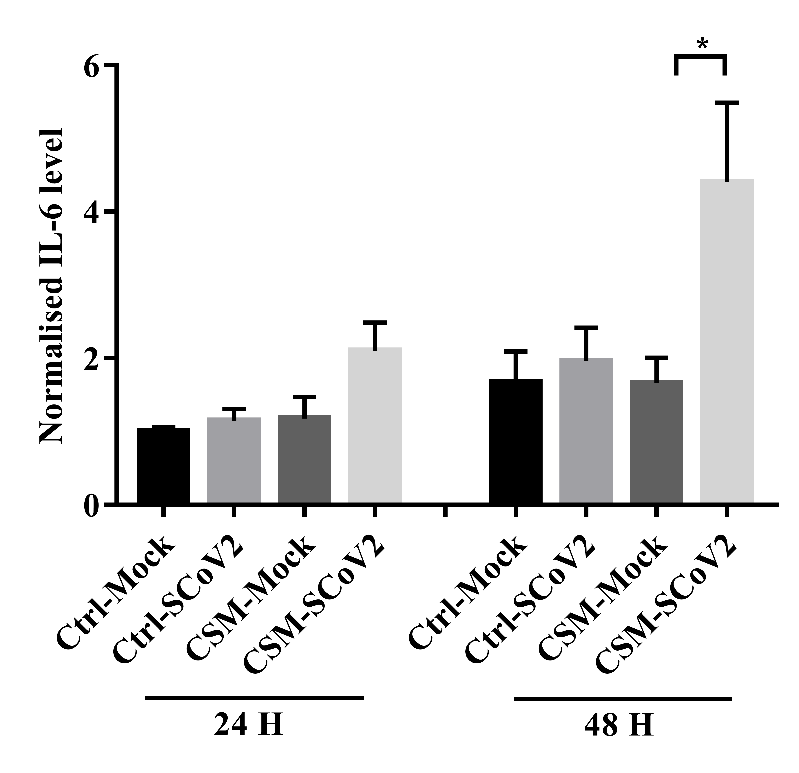


**Fig. S3** Effect of cigarette smoke on SARS-CoV-2-induced cytokine IL-6 release at the apical side of the supernatants collected from cell cultures. Ctrl, control; CSM, cigarette smoke medium; SCoV2, SARS-CoV-2; H, hours. Values are expressed as mean ± SEM (n=5). ^*^*p*<0.05 for One-way ANOVA test with post hoc analysis and Tukey correction.

**Table S1** Quantitative PCR primer sequences

| **Target gene** | **Primer sequences (5’-3’)** |
| --- | --- |
| Angiotensin converting enzyme 2 (ACE2, total) | Forward: TGGGACTCTGCCATTTACTTAC  Reverse: CCCAACTATCTCTCGCTTCATC |
| ACE2 (long form) | Forward: CAAGAGCAAACGGTTGAACAC  Reverse: CCAGAGCCTCTCATTGTAGTCT |
| ACE2 (short form) | Forward: GTGAGAGCCTTAGGTTGGATTC  Reverse: TAAGGATCCTCCCTCCTTTGT |
| E-cadherin | Forward: CCCACCACGTACAAGGGTC  Reverse: CTGGGGTATTGGGGGCATC |
| Forkhead box J1 (FOXJ1) | Forward: TCGTATGCCACGCTCATCTG  Reverse: CTTGTAGATGGCCGACAGGG |
| Interferon (IFN)-β | Forward: CAACTTGCTTGGATTCCTACAAAG  Reverse: TGCCACAGGAGCTTCTGACA |
| Interferon-stimulated gene  (ISG) 15 | Forward: CAAATGCGACGAACCTCTGA  Reverse: CCGCTCACTTGCTGCTTCA |
| Interleukin (IL)-6 | Forward: ACATGTGTGAAAGCAGCAAAG  Reverse: TGATGATTTTCACCAGGCAAGT |
| IL-8 | Forward: GAGAGTGATTGAGAGTGGACC  Reverse: ACTTCTCCACAACCCTCTGC |
| IL-28 | Forward: TTTAAGAGGGCCAAAGATGC  Reverse: TGGGCTGAGGCTGGATACAG |
| IL-29 | Forward: GCCCCCAAAAAGGAGTCCG  Reverse: AGGTTCCCATCGGCCACATA |
| Kinesin-like protein 27 (Kif27) | Forward: AGCTTGCCTGAGTCCTGTTGA  Reverse: GCTTCTCGCAAATTCACCACC |
| Myxovirus resistance protein 1 (MxA) | Forward: GAGGCCAGCAAGCGCAT  Reverse: TGGAGCATGAAGAACTGGATGA |

**Table S1** Quantitative PCR primer sequences (Continued)

| **Target gene** | **Primer sequences (5’-3’)** |
| --- | --- |
| Mucin 5AC (MUC5AC) | Forward: CTTTGGCATCTGTGAGGAGC  Reverse: CACAGAAGCAGAGGTCTTGC |
| Ribosomal protein S13 (RPS13) | Forward: CTTTACCCTATCGACGCAGC  Reverse: TCTGTGAAGGAGTAAGGCCC |
| SARS-CoV-2, open reading frame 1b (ORF1b) | Forward: TGGGGYTTTACRGGTAACCT  Reverse: AACRCGCTTAACAAAGCACTC |
| Serine/Threonine kinase 36 (STK36) | Forward: CAAGGGGTGACCTTTGACC  Reverse: GGTGGAGTCAACCGAACCT |
| Transmembrane serine protease (TMPRSS) 2 | Forward: CAGACCAGGAGTGTACGGGAAT  Reverse: TCTGCCCTCATTTGTCGATAAA |
| TMPRSS4 | Forward: CCAAGGACCGATCCACACT  Reverse: GTGAAGTTGTCGAAACAGGCA |
| Tumor necrosis factor α (TNF-α) | Forward: GCAGGTCTACTTTGGGATCATTG  Reverse: GCGTTTGGGAAGGTTGGA |
| Zonula occluden-1 (ZO-1) | Forward: GCGGTCAGAGCCTTCTGATC  Reverse: CATGCTTTACAGGAGTTGAGACAG |
